# Ovarian hyperstimulation syndrome and pregnancy luteoma mimicking malignant ascites: a rare case report

**DOI:** 10.1186/s13048-023-01186-2

**Published:** 2023-05-16

**Authors:** Jing Chen, Qian Sun, Tao Zhu, Kezhen Li

**Affiliations:** Department of Obstetrics and Gynecology, Tongji Hospital, Tongji Medical College, Huazhong University of Science and Technology, 1095 Jiefang Ave. Wuhan, Hubei, 430030 China

**Keywords:** OHSS, Pregnancy luteoma, Malignant ascites, CA125, Case report

## Abstract

**Background:**

During pregnancy, both ovarian hyperstimulation syndrome (OHSS) and pregnancy luteoma could manifest as massive ascites, enlarged ovaries, or elevated serum levels of cancer antigen 125 (CA125), and atypical cells may be found in the ascitic fluid of OHSS patients. Whether this should be treated aggressively as peritoneal carcinomatosis is controversial.

**Case presentation:**

A 35-year-old G2P1A1 woman with secondary infertility had a successful pregnancy after one cycle of assisted reproductive technology. The patient complained of lower abdominal distension, oliguria, and poor appetite 19 days after embryo transplantation. She was diagnosed with late-onset OHSS. Although the size of the ovaries decreased bilaterally to the normal range at 12 weeks of gestation after prompt medical care, the ascites increased again after an initial decreasing trend. Elevated serum levels of CA125 (191.1 IU/mL), and suspected adenocarcinoma cells were observed in the ascitic fluid. Although further magnetic resonance imaging examination or diagnostic laparoscopy was recommended, the patient was provided with supportive treatment and closely monitored upon her request. Surprisingly, her ascites diminished, and serum level of CA125 started to decline at 19 weeks of gestation. During cesarean section, pathological examination of the solid mass in the right ovary revealed pregnancy luteoma, which was presumably the other cause of the intractable ascites.

**Conclusions:**

Caution should be exercised in cases of suspicious malignant ascites during pregnancy. This may due to OHSS or pregnancy luteoma, in which abnormalities usually regress spontaneously.

## Background

Ascites refers to the pathological accumulation of excess fluid in the peritoneal cavity. It is a clinical problem characterized by diverse pathogenesis and a broad range of medical disorders, that require an in-depth differential diagnosis. Malignant ascites, namely malignancy-induced ascites, is the second most common cause of ascites, accounting for 10% of all cases [1]. From the perspective of obstetric and gynecological diseases, ovarian hyperstimulation syndrome (OHSS), an iatrogenic complication caused by an uncontrolled response to controlled ovarian stimulation, is the most common cause of ascites, with an incidence of moderate-to-severe OHSS being 3–10% of the cycles of assisted reproductive technology [2]. The pathophysiological mechanisms underlying the development of ascites vary in different diseases. Malignant ascites can be caused by conditions such as hypoproteinemia, impaired lymphatic drainage, or increased vascular permeability [3]. In OHSS, increased vascular permeability leads to a shift in fluid from the intravascular volume to the extravascular space, resulting in subsequent fluid accumulation, especially in the abdominal cavity [4, 5]. An accurate diagnosis is a prerequisite for appropriate and successful treatment. Ascitic fluid cytology is an essential examination that should be performed for the differential diagnosis of malignant ascites in which positive cytological findings are highly suggestive of malignancy. Depending on pathogenesis, the ascitic fluid contains variable proportions of suspended cells, and determining cellular composition is helpful in determining the cause of ascites. In malignant ascites, the cellular population consists of different proportions of tumor cells, mesothelial cells, fibroblasts, macrophages, white blood cells and red blood cells [6]. Ascitic fluid cytology has a sensitivity of 83%, and can be as high as 97% if three separately obtained samples are analyzed [7]. Misdiagnosis of benign neoplasms due to ascitic cytology is rare. Given its minimal invasiveness, repeatability, and feasibility, ascitic fluid cytology has been widely used for the diagnosis of malignant ascites, especially for the preoperative differential diagnosis of ovarian cancer and benign ovarian tumors. However, atypical cells in ascitic fluid cytology are usually detected in cases of severe OHSS, although these patients exhibit no evidence of ovarian cancer during long-term follow-up [8]. Whether this should be treated aggressively as peritoneal carcinomatosis is controversial in women with OHSS and suspicious tumor cells on ascitic fluid cytology.

Herein, we report a case of OHSS following ovulation induction therapy. The patient presented with massive ascites, enlarged ovaries, elevated serum levels of cancer antigen 125 (CA125), and suspected adenocarcinoma cells in the ascitic fluid that mimicked malignant ascites. However, the abnormalities regressed spontaneously, and pathological examination of the right ovary revealed a pregnancy luteoma during cesarean section. This was an unusual case of OHSS accompanied by a pregnancy luteoma that masqueraded as malignant ascites.

## Case presentation

**Fig. 1:**
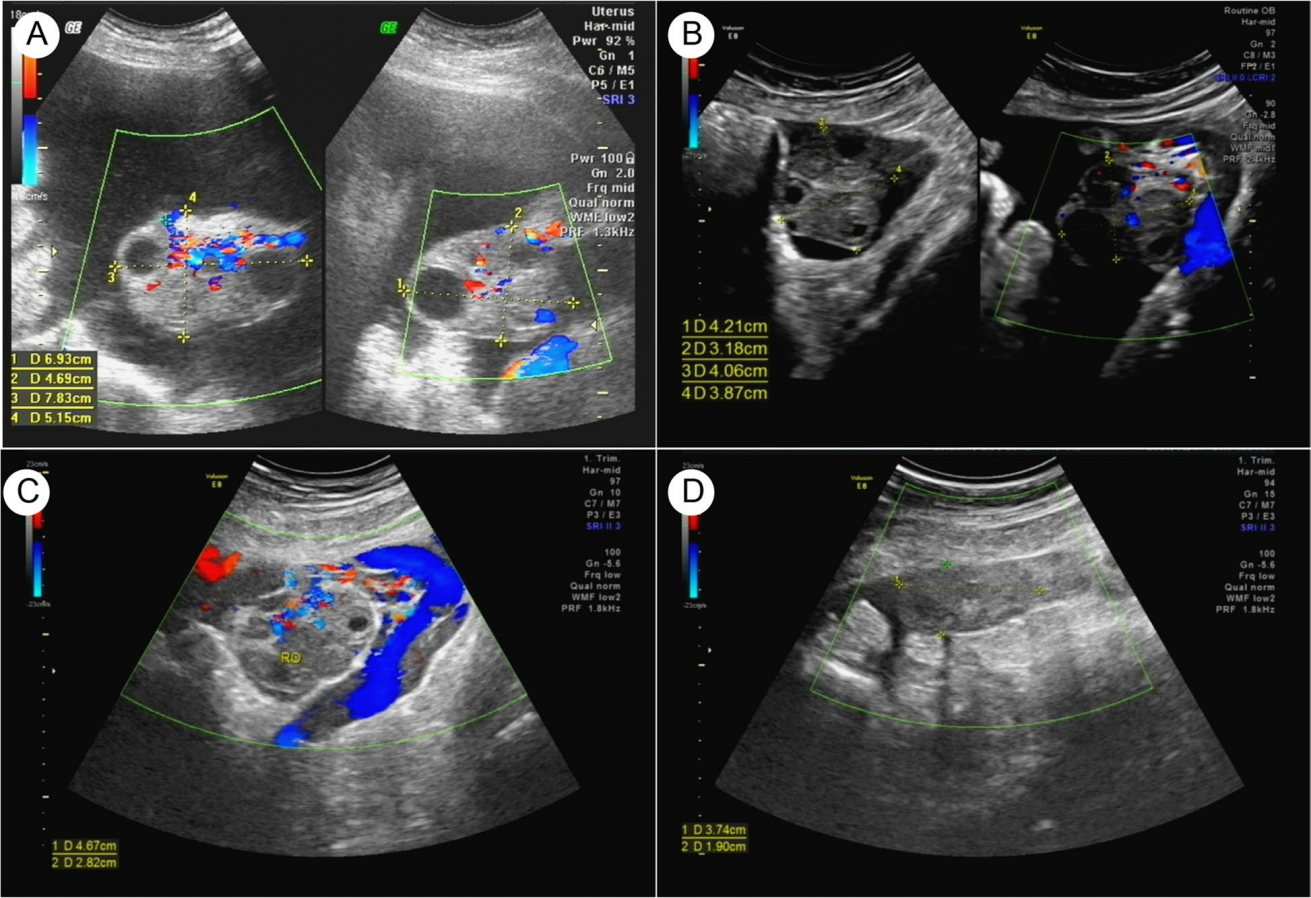
Ultrasound images of the ovaries. **A** Enlarged bilateral ovaries (left, 6.9 × 4.7 cm; right, 7.8 × 5.2 cm) with multiple anechoic areas were detected at five weeks of gestation. **B** At the 15 weeks of gestational age, the right ovary became solid, and multiple cysts involved the left ovary. At 24 weeks of gestational age, there was right (**C**) and left (**D**) ovarian enlargement, with hypoechoic area in the parenchyma and visible rich blood flow signals in the right ovary

**Fig. 2:**
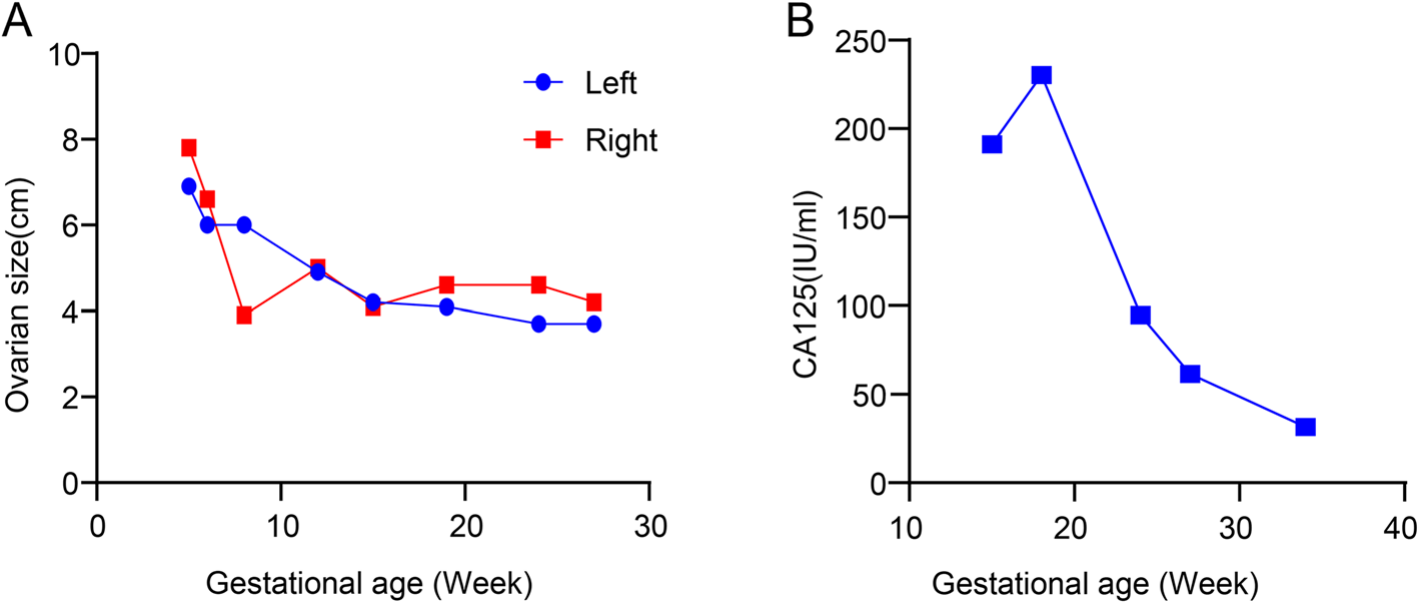
Dynamic change in (**A**) ovarian size (cm) and (**B**) serum CA125 level (IU/mL)

A 35-year-old G2P1A1 woman with male factor infertility successfully became pregnant after undergoing a cycle of assisted reproductive technology. Her husband had asthenospermia, and intra-uterine insemination attempts were unsuccessful. Gonadotropin-releasing hormone-agonist (GnRH-agonist) long-protocol was used for controlled ovarian hyperstimulation: triptorelin was administered daily in the mid-luteal phase. Fourteen days later, gonnadotrophin was initiated daily, and the GnRH-agonist was continued until triggered by human chorionic gonadotropin (hCG). Thirty-six hours after the ovulatory trigger, fourteen eggs were collected. After in vitro fertilization, two 3-day-old embryos were used for fresh embryo transfer. The procedure was uneventful, with no discomfort or symptoms. The patient complained of lower abdominal distension, oliguria, and poor appetite 19 days after the embryo transplantation. After five days, she visited the hospital and a grossly distended abdomen and shifting dullness were observed upon physical examination without tenderness, rebound tenderness, or a palpable liver and spleen. Other medical history, such as hepatitis, liver cirrhosis, cancer, or family history of cancer were unremarkable. Enlarged bilateral ovaries (left, 6.9 × 4.7 cm; right, 7.8 × 5.2 cm) with multiple anechoic areas were detected (Fig. 1) in the pelvic cavity using B-mode ultrasonography, accompanied by large-volume ascites in both abdominal (left, 17.7 cm; right, 13.7 cm) and pelvic cavities (8.5 cm). Her 24-h urine output was 500–700 mL/day. Blood test results showed elevated white blood count (14.26 × 10^9^/L), elevated levels of hematocrit (41.6%), D-dimer (4.44 µg/mL FEU) and liver enzymes (alanine aminotransferase, 67 U/L; alanine aminotransferase, 100 U/L), with decreased serum albumin level (30.3 g/L) and hyponatremia (132.00 mmol/L). The patient was admitted with a presumed diagnosis of severe OHSS [9] and she underwent symptomatic treatment in the general ward with intravascular perfusion (Dextran-40 10%, 500 mL; intravenous drip, q.d.) with adequate urine output, complement albumin, and prophylactic anticoagulation. Abdominal paracentesis and intraperitoneal drainage of ascitic fluid were performed several times to relieve abdominal distension and oliguria. A week later, her enlarged ovaries began to decrease in size, but she continued having massive ascites despite a brief decline (Fig. 2). All treatments and procedures were performed with informed consent from the patient.

**Fig. 3:**
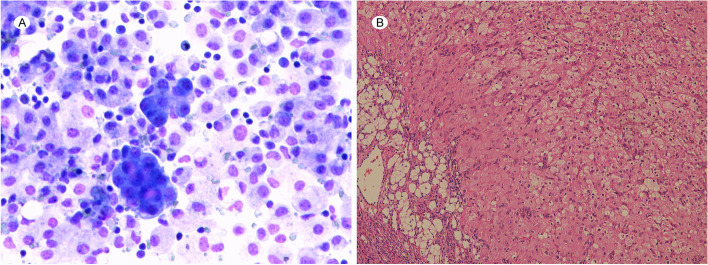
Results of ascitic fluid cytology and histopathological tests. **A** Cytological examination of cells in the ascitic fluid (magnification 400 ×). **B** Microscopic examination of right ovarian mass (magnification 400 ×)

**Fig. 4:**
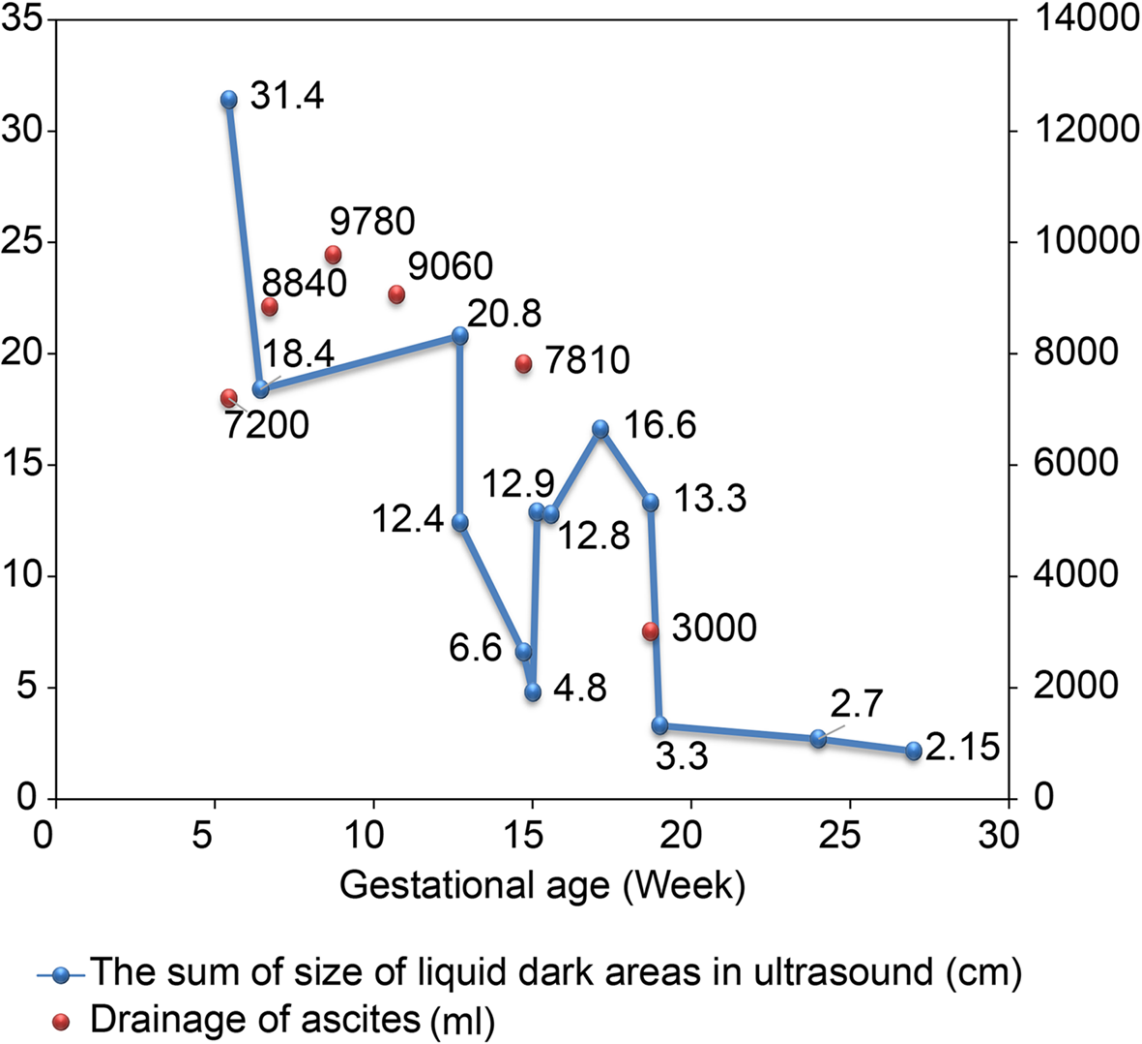
Dynamic change in ascites

Since symptomatic and supportive care for OHSS did not alleviate the patient's symptoms, at 15 weeks of gestation, serum tumor marker tests and cytological examination of the ascitic fluid specimen were performed to explore the cause of the ascites. This revealed high serum level of CA125 (191.1 IU/mL) and alpha fetoprotein (AFP, 34.21 ng/mL). Carcinoembryonic antigen (CEA) levels were within normal limits during pregnancy. Ultrasonography revealed that the right ovary was solid, but multiple cysts were present in the left ovary. Microscopically, a large number of mesothelial cells, numerous lymphocytes, and a few suspected cancer cells were observed on cytological examination (Fig. 3). At 19 weeks of gestation, a second analysis of ascitic fluid specimens revealed suspected adenocarcinoma cells and high CA125 level (2471.6 IU/mL). Simultaneously, the serum CA125 level increased to 230.3 IU/mL. Even though we explained to the patient that there were no reported adverse maternal or fetal effects from magnetic resonance imaging (MRI) during pregnancy, the patient declined MRI because of the daunting nature of the imaging examination and cost involved. After discussing treatment options and the need and risks of surgical exploration with the patient, the patient and her family decided to continue the pregnancy with frequent visits to the outpatient department although further evaluation or laparoscopic exploration was strongly recommended. After 19 weeks of gestation, the ascites gradually decreased after peritoneal drainage of 3000 mL of ascitic fluid (Fig. 4), and the serum level of CA125 gradually decreased to the recommended range between 19–34 weeks of gestation (Fig. 2). Subsequently, she underwent a prenatal examination as scheduled without any abnormalities. A post-hoc review of ultrasound at 24 weeks of gestational age revealed bilateral ovarian enlargement, with 2 × 3-cm hypoechoic areas in the parenchyma and visible rich blood flow signals (Fig. 1C and D).

At 35 weeks and 2 days of gestation, regular contractions were detected in the patient, with 2 cm cervical dilation. A diagnosis of preterm labor was considered. However, she had a twin pregnancy with a previous cesarean section. Considering the possibility of an ovarian tumor, an emergency cesarean delivery was performed upon the patient’s request. Normal twins, one female and one male, as examined by a pediatrician, were delivered. The female infant showed no evidence of virilization. Intraoperative, the uterus and left ovary appeared macroscopically normal, whereas an enlarged right ovary was observed, which was generally hard, with nodules on palpation. A wedge resection of the right ovary was performed. The final pathological examination revealed pregnancy luteoma, which is an extremely rare ovarian tumor. Macroscopic examination of the ovarian biopsy tissue showed a circumscribed, firm, golden-brown lesion with a grayish-white nodule measuring 2 cm. Microscopically, paraffin‑embedded sections of the right ovarian mass showed a lesion composed of cells arranged in a trabecular or follicular pattern with stromal cell proliferation. The cells were polygonal or irregular in shape, had abundant cytoplasm, and were filled with eosinophilic granular cells and lightly or undyed lipid cells. The follicular structure contained eosinophilic colloids scattered throughout the lesion, and the interstitium was composed of small parenchymatous vessels and fine fibrous tissues (Fig. 3). A pathological diagnosis of pregnancy luteoma was made based on the pregnancy status and histopathological features. No examination for androgens was performed during the pregnancy, and further inquiries about her medical history revealed no virilization with androgen elevation, including hirsutism, acne, or deepening of the voice.

At five years of close follow-up after delivery, ultrasonography and tests for serum tumor markers and sex hormones yielded no abnormal results. To date, the patient had no recurrence of ascites, elevated CA125 levels, or adnexal masses.

## Discussion and conclusions

OHSS is a common but severe and potentially life-threatening iatrogenic complication usually observed in women undergoing ovarian stimulation procedures during infertility treatment. It is characterized by ovarian enlargement, multiple serous cavity effusions, elevated estradiol levels, and thromboembolism [10]. According to clinical presentation period, this syndrome is classified as early- or late-onset OHSS [11]. Early-onset OHSS occurs within 9 days of hCG administration, as a consequence of an ovarian hyperresponse to exogenous hCG. However, late-onset OHSS, can be observed at > 10 days after a trigger and is related to endogenous hCG [11]. Endogenous hCG, which is produced by a conceptus, induces the production of vascular endothelial growth factor (VEGF) and VEGF receptor-2 by granulosa-lutein cells, which directly increase vascular permeability [12, 13]. The clinical course of late-onset OHSS is generally < 2 months, and the maximum hospital stay is 65 days from previous published literatures [14]. In our case, the patient showed long-term ascites accumulation at 19 weeks of gestational age, although the size of the bilateral ovaries decreased to the normal range with an obscure cystic lesion, and the serum hCG level showed a significant decrease. Therefore, in the second trimester, intractable and persistent ascites cannot be considered a symptom of OHSS, but is caused by a combination of multiple mechanisms.

Ultrasonography has limited efficiency in screening for morphological changes in the ovaries during pregnancy due to an enlarged uterus. Thus, we observed only a slight transient enlargement of the right ovary at 15 weeks of pregnancy, without obvious adnexal mass or external excrescences. Because the patient refused to undergo MRI, ascitic fluid cytology and serum tumor biomarkers were evaluated. The combination of massive ascites, enlarged ovaries, elevated CA125 levels, CA125/CEA ratio, and suspected adenocarcinoma cells in the ascitic fluid indicated ovarian malignancy. Therefore, diagnostic laparoscopy was recommended for targeted biopsy and histological evidence.

These facts proved that we had been confused by the results of the ascites cytology and serum tumor markers. The presence of tumor cells in the ascitic fluid is usually suggestive of malignancy; however, previous literatures have also reported the presence of atypical cells in the ascitic fluid of women with severe OHSS [8, 15]. In the present case, we observed suspected adenocarcinoma cells twice, although the possibility of malignancy was ruled out. Additionally, CA125, a biomarker most often used for the diagnosis of epithelial ovarian cancer, can increase in the first trimester, with a reported maximum value of up to 550 IU/mL [16]. Therefore, CA125 has limited diagnostic capacity for ovarian malignancy. However, the ratio of CA125 to CEA has diagnostic implications for ovarian epithelial carcinoma. Primary ovarian cancer was indicated in patients with a CA125/CEA ratio > 25 [17]. However, its value in pregnant women remains unclear. Additionally, AFP is an unreliable tumor marker, because it is elevated during pregnancy, and abnormal levels are often indicative of pregnancy complications or birth defects [16]. Therefore, the diagnostic utility of suspicious cells in the ascitic fluid and tumor markers in pregnant women must be carefully considered, especially in patients diagnosed with OHSS. Caution regarding radical surgical intervention is needed. Surgical intervention is indicated if suspicion of malignancy or an adnexal mass > 10 cm due to the increased risk of complications. Features of adnexal masses, such as solid components, papillary projections, and multiple septae, increase the likelihood of malignancy [18].

During the cesarean section, pathological examination of the right ovary revealed a pregnancy luteoma, which was presumed to be other cause of intractable ascites. Pregnancy luteoma is a rare hormone-dependent benign ovarian neoplasm that develops during pregnancy [19]. It usually has no clinical symptoms and is often detected incidentally during routine examinations or cesarean sections. Affected women may present with bilateral solid ovarian mass, elevated serum testosterone levels and symptoms of virilization [20]. Given the solid nature of the mass, it is difficult to differentiate pregnancy luteomas from other solid ovarian neoplasms, including luteinized thecomas, granulosa cell tumors and Leydig cell tumors. The bilateral solid masses, elevated total testosterone and dehydroepiandrosterone sulfate levels supported the diagnosis of pregnancy luteoma. Elevated levels of tumor markers, including CA125, inhibin, AFP and β-hCG, can help in the differential diagnosis of ovarian neoplasms, but remain uncertain in pregnant patients. Due to the absence of virilization in our case and the confusability of OHSS in early pregnancy, a prompt and accurate diagnosis of pregnancy luteoma was missed. Diagnostic and management challenges related to pregnancy luteoma have been reported because it can be confused with malignant ovarian tumors, thereby leading to unnecessary oophorectomies during pregnancy. Accurate diagnosis is of great value for both mothers and children.

Pregnancy luteoma with massive ascites are rare. Three cases of luteoma of pregnancy presenting with massive ascites and elevated serum CA125 levels have been reported. The quantity of ascitic fluid ranges between 1000–8500 ml. Exploratory laparotomy has been performed in all reported cases for considering ovarian malignancy [21–23]. In contrast, in our case, massive ascites and CA125 levels showed spontaneously normalized by unknown mechanisms after 19 weeks of gestation. Furthermore, massive ascitic fluid with suspected adenocarcinoma cells and elevated CA125 levels during pregnancy resembled the symptoms of malignant tumors, which have rarely been reported in pregnancy luteoma.

Although this was an unusual case of OHSS accompanied by pregnancy luteoma that masqueraded as malignant ascites, the patient was managed appropriately by both the clinician and patient and was spared from unnecessary oophorectomy. This case can be generalized to a larger population, including intractable ascites in patients with OHSS and pregnancies of unknown location. However, this case report has some limitations. Due to its retrospective nature, examinations for androgens, human epididymis protein 4 and MRI during pregnancy were not conducted, therefore, the diagnosis of pregnancy luteoma relied on a pathological examination. Additionally, the cause of suspected carcinoma cells in the ascitic fluid remains unknown. We expect that in the future, detailed studies will address the pathomechanism underlying the presence of atypical cells in the ascitic fluid of patients with OHSS.

In conclusion, during pregnancy, OHSS and pregnancy luteoma both could manifest as massive ascites, enlarged ovaries, and elevated serum levels of CA125, and atypical cells may be founded in the ascitic fluid. These clinical features mimic those of malignant ascites; however, surgical intervention should be considered to avoid adverse pregnancy outcomes. Conservative management and supportive treatment are appropriate when suspicious malignant ascites arise from OHSS or pregnancy luteoma because these abnormities usually regress spontaneously.

## Data Availability

The datasets used and analyzed in the current study are available from the corresponding author upon reasonable request.

## Abbreviations

AFP: Alpha fetoprotein
CA125: Cancer antigen 125
CEA: Carcinoembryonic antigen
hCG: Human chorionic gonadotropin
MRI: Magnetic resonance imaging
OHSS: Ovarian hyperstimulation syndrome
VEGF: Vascular endothelial growth factor
